# Pneumomediastinum in Vaping-Associated Acute Lung Injury: An Unusual Presentation

**DOI:** 10.7759/cureus.45772

**Published:** 2023-09-22

**Authors:** Olawale Babalola, Maureen Ikedinobi, Venkatkiran Kanchustambham

**Affiliations:** 1 Pulmonology, University of North Dakota School of Medicine and Health Sciences, Fargo, USA; 2 Pulmonary Critical Care, University of North Dakota, Fargo, USA

**Keywords:** e-cigarette or vaping product use-associated lung injury (evali), ground glass opacity, subcutaneous emphysema, secondary pneumomediastinum, centers for disease control and prevention (cdc)

## Abstract

The use of vaping products among adolescents continues to be on the rise despite known health risks. As a result, there are increasing cases of E-cigarette or vaping product use-associated lung injury (EVALI) across the United States especially among male Caucasian users of vaping products. The clinical presentation of EVALI follows the classic pattern of acute lung injury; however, there are peculiar cases with unusual symptomatology and radiographic findings. In this report, we present a case of a 25-year-old male with hemoptysis, subcutaneous emphysema, and pneumomediastinum in the setting of EVALI. He was treated with nebulized tranexamic acid and methylprednisolone with the resolution of symptoms. The diagnostic workup and management of suspected EVALI are discussed in detail. This case highlights how EVALI can present in an atypical manner and why clinicians must be cognizant of the variations in manifestations in order to facilitate early management. Overall, this case further highlights the need for clinicians to continuously push against the use of vaping products in the adolescent group, given that the occurrence of acute lung injury at a younger age predisposes to early-onset chronic lung disease.

## Introduction

There was an outbreak of E-cigarette or vaping product use-associated lung injury (EVALI) in the United States following the introduction of E-cigarettes to the country in 2007. Consequently, in 2018, the use of E-cigarettes among adolescents was declared an epidemic [1]. Regardless of the national attention and massive funding allocated to research in this area, vaping among adolescents and youths continues to be on the rise [1]. Of all the E-liquids available in the market, tetrahydrocannabinol (THC) E-liquids are the most reported E-liquids associated with EVALI [2,3]. An analysis from the New York State Department of Health in 2019 announced vitamin E acetate (VEA) as the primary component of THC E-liquids [2]. However, it remains unclear if VEA mediates all the clinical findings seen in patients with EVALI. Adolescents are mainly affected, specifically male Caucasians between ages 18 and 24 years old [2]. A case series from Illinois and Wisconsin reported male Caucasians within the age range of 15-53 years as those commonly affected by EVALI [3]. As of October 22, 2019, 34 deaths from EVALI have been reported to the Centers for Disease Control and Prevention (CDC). Of the 29 deaths among patients with EVALI, 59% were male with a median age of 45 years [4]. The typical presentation of EVALI includes shortness of breath, cough, fever, chills, vomiting, and ground glass opacities on chest imaging. In some severe and atypical cases, patients may present with pneumothorax and pneumomediastinum. The prompt identification of an unusual presentation of EVALI allows a clinician to initiate early management and, overall, reduces health burden.

## Case presentation

We present a case of a 25-year-old male who had experienced a fall-related syncopal episode followed by recurrent cough with six episodes of about a teaspoonful of frank red sputum and shortness of breath two days prior to presenting to the emergency department. Past medical history is significant for bipolar affective disorder, seizure-like activities, and benzodiazepine abuse. He is a chronic daily vape user, and he could not particularly remember how long he has been vaping but estimated about 5-7 years. The main vape content is THC, but he did admit that there are possibilities of the vape being laced with other substances such as fentanyl. He denies inhaled substance use. He denies fever or chills and has no known drug or medication allergies and no family history of lung disease. The patient works in retail and denies exposure to occupationally hazardous substances.

Vital signs on presentation were a temperature of 97.8 degrees Fahrenheit, pulse rate of 129 beats per minute, blood pressure of 125/82 mmHg, respiratory rate of 22 breaths per minute, and oxygen saturation of 94% on room air. Significant physical assessment findings include scattered crackles and wheezing in all lung fields on auscultation. There was crepitus spanning from the right superior chest wall to the lower one-third of the right lateral neck region.

**Figure 1:**
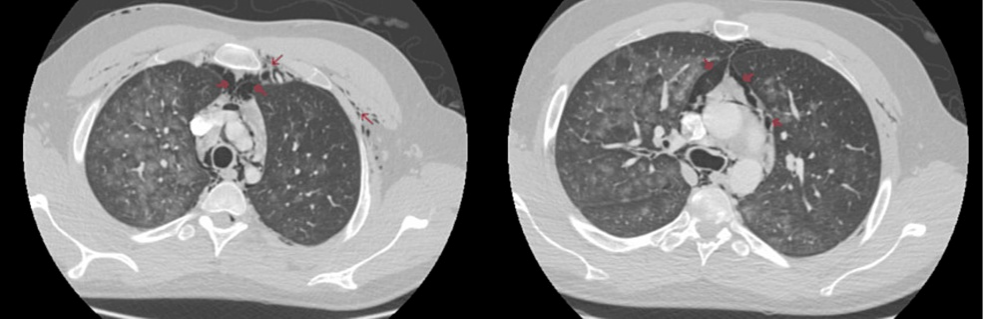
CT of the chest showing areas of air collection within the mediastinum.

**Figure 2:**
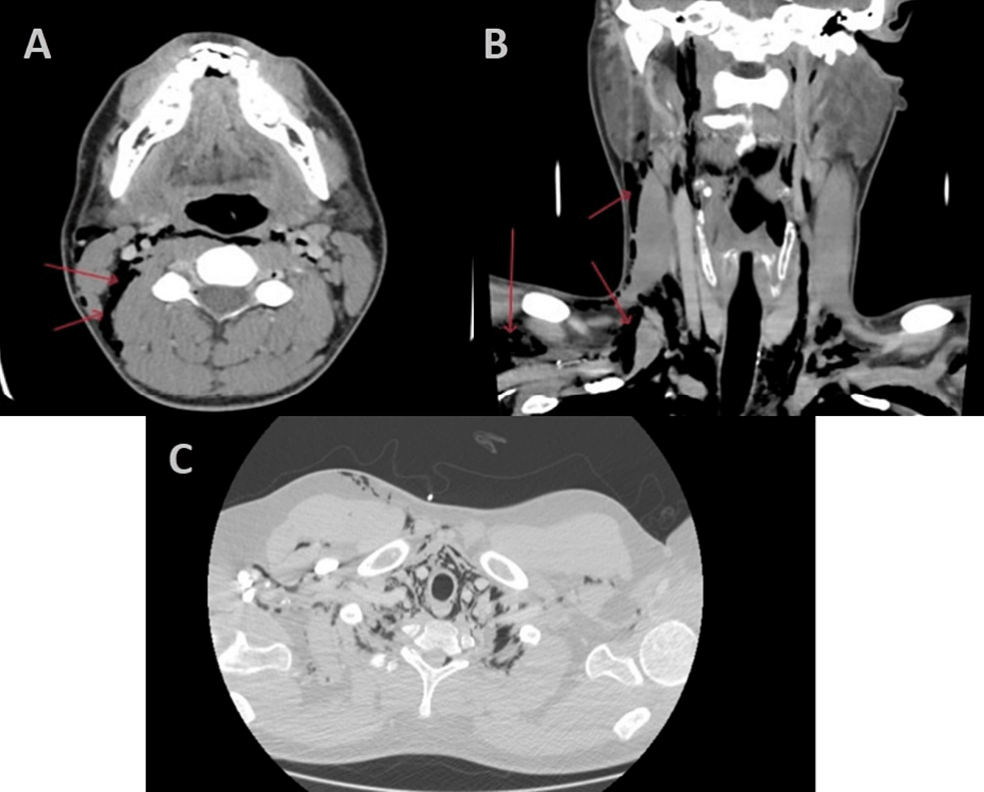
CT showing air collection within the soft tissue of the neck.

**Figure 3:**
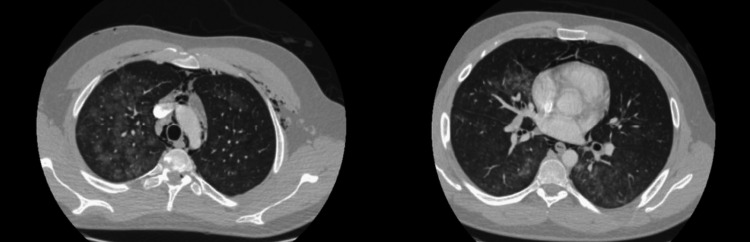
Diffuse ground glass opacities on a CT of the chest of a chronic vape user.

Chest X-ray was obtained, which showed pneumomediastinum and subcutaneous emphysema overlying the patient's lower neck region. This was further evaluated with a CT of the chest and neck, which confirmed the X-ray findings and, in addition, revealed the presence of diffuse ground glass airspace opacities (Figures 1-3). A CT of the head was also obtained due to a history of fall; no acute intracranial process was identified. Given the degree of subcutaneous emphysema and pneumomediastinum, an evaluation to rule out an esophageal source was conducted. Fluoroscopic single-contrast esophageal imaging using water-soluble contrast identified no leak in the esophagus.

Laboratory results were notable for leukocytosis with WBC count of 16.7 K/μL (reference range: 4.0-11.0 K/μL). There was mild transaminitis with aspartate aminotransferase (AST) of 200 U/L (reference range: 5-34 U/L) and alanine transaminase (ALT) of 121 U/L (reference range: 0-55 U/L). C-reactive protein (CRP) and procalcitonin were elevated at 223.6 mg/L (normal: ≤5.0 mg/L) and 14.86 ng/mL (normal: <0.07 ng/mL), respectively. Blood and sputum cultures were obtained, and a viral panel for SARS-CoV-2 and influenza returned negative. Qualitative Fungitell, *Legionella*, and strep pneumonia urine antigens also returned negative. Rapid drug screen was positive for THC and fentanyl.

Given the presence of leukocytosis and elevated inflammatory markers, there were concerns for infectious or autoimmune etiology. The patient was hospitalized and started on azithromycin and ceftriaxone to cover for possible community-acquired pneumonia. Autoimmune panel was obtained, and pulmonology was consulted. Antinuclear antibody (ANA) and antineutrophil cytoplasmic antibody (ANCA), proteinase 3 (PR3) and myeloperoxidase (MPO), returned negative. On review by pulmonology, vaping-associated lung injury was suspected as the most likely etiology given the patient's vaping history and characteristic CT findings with the presence of unprovoked pneumothorax/pneumomediastinum. HIV screening was recommended, which returned negative.

Treatment

He was started on continuous oxygen therapy via non-rebreather mask with scheduled nebulized tranexamic acid at least three times daily. Other nebulizer treatments include albuterol, ipratropium, and Pulmicort. Methylprednisolone was initiated at 1 mg/kg/day in two divided doses. He was transitioned on day 3 to oral prednisone at 0.5 mg/kg/day for a total of seven days. Antibiotics were discontinued as blood cultures yielded no growth and no positivity for any organisms. The symptoms gradually resolved with no episode of hemoptysis reported on day 3 of hospitalization, and laboratory values returned within normal range. He was subsequently discharged after education and counseling on the effect of vaping on lung health.

Follow-up

**Figure 4:**
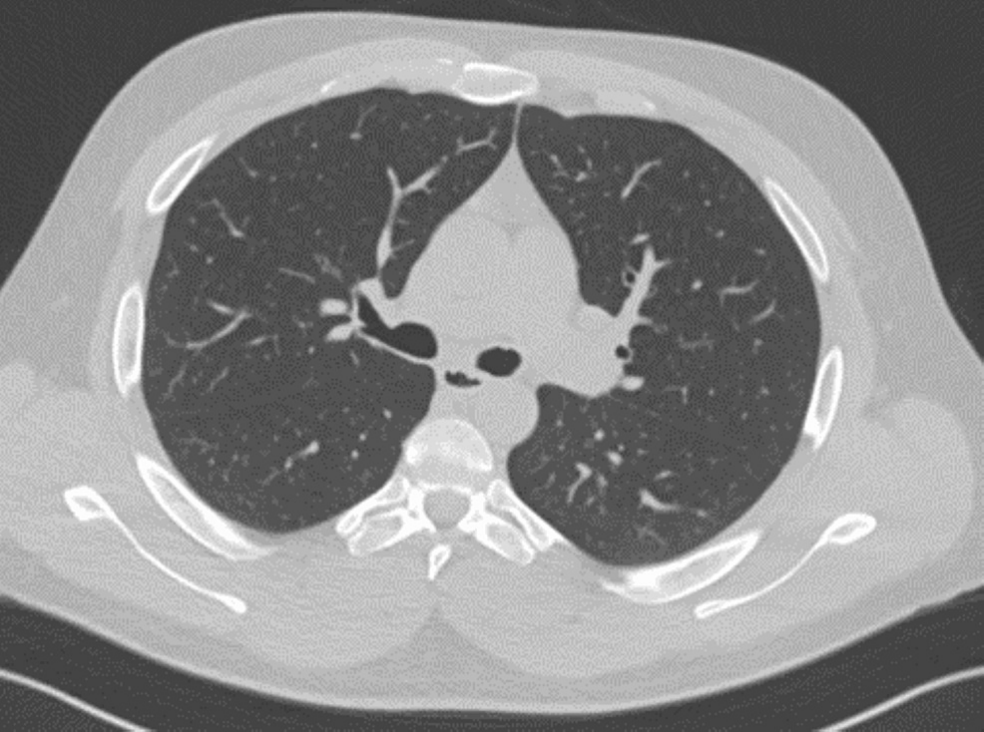
Repeat CT of the chest during four-week follow-up showing the complete resolution of pneumomediastinum and ground glass opacities.

He presented four weeks after discharge to the pulmonology department for follow-up. Repeat CT showed the complete resolution of previous findings (Figure 4). He was advised to continue abstaining from vaping and started on varenicline. A referral was made to Quitline for further substance use management.

## Discussion

The pathophysiology of EVALI remains unknown. However, ongoing research has highlighted the crucial constituents of E-cigarette and E-liquids that can contribute to the clinical features seen in patients that present with EVALI. Currently, no direct causal relationships have been established due to the heterogeneity of the chemical composition used in E-cigarette [2]. In addition, the chemical constituents in the E-liquids undergo heat transformation through the E-cigarette heating coil to produce a different compound with a distinct toxic profile [3]. A recent review article by Winnicka and Shenoy described an increase in the concentration of cytokines such as interleukin (IL) 6, monocyte chemotactic protein-1 (MCP-1), IL-1 alpha, and IL-13 in mice exposed to E-cigarette vapor [5]. This strongly confirms the suspicion of E-cigarette and E-liquid leading to inflammatory changes seen in the lungs and systemically.

Patients with EVALI can present with a variety of clinical symptoms, which include respiratory, gastrointestinal, and constitutional symptoms [2,3]. Characteristically, patients present with respiratory symptoms such as hemoptysis, cough, and shortness of breath. Vital signs on initial presentation are usually abnormal with the presence of fever, tachypnea, tachycardia, and hypoxemia seen in most cases [2,3]. In addition, laboratory findings usually reveal the presence of an ongoing inflammatory process with elevated leukocytes, erythrocyte sedimentation rate (ESR), and procalcitonin. Mild transient elevated liver function enzymes can also be seen.

According to the CDC criteria for severe pulmonary disease associated with E-cigarette, EVALI should be highly suspected if the patient has a history of vaping in the past 90 days; the absence of alternative plausible diagnosis such as cardiac, rheumatologic, or neoplastic process; the absence of pulmonary infections; and the presence of pulmonary infiltration on imaging. In addition, the CDC guideline recommends chest radiography in any patient presenting with a history of vaping regardless of the presence or absence of respiratory or gastrointestinal symptoms [4]. Only a few cases of EVALI have been reported to be associated with pneumomediastinum and subcutaneous emphysema. In a case series reported by Messina et al., the radiologic findings of the six patients with EVALI did not show pneumomediastinum [6]. In another case series by Layden et al., of the 91 patients with EVALI who had CT imaging done, pneumomediastinum was found in only six patients [3]. Ground glass pulmonary opacifications with or without subpleural sparing are the most common CT findings seen in the cases of EVALI [2]. Pneumothorax has been reported in other cases of EVALI and is considered a probable radiologic finding in EVALI [5]. It is advised to report cases of lung injury of unknown cause and a history of vaping within 90 days to the local health department [7]. The role of bronchoscopy and lung biopsy remains undefined in the diagnosis of EVALI and was not utilized in our case.

EVALI is a diagnosis of exclusion, and as a result, it takes several days for other etiologies to be ruled out. However, within the time frame of ruling out infectious etiologies, most patients are started on antibiotics, due to the presence of leukocytosis, abnormal vital signs, and constitutional symptoms seen with presentation. Corticosteroid is the mainstay treatment for EVALI, even though no clinical trial supports this consensus [2]. Corticosteroid dose and duration are determined by clinicians depending on the severity of the case. In some cases, patients with EVALI may require oxygen supplementation, noninvasive ventilation, and intubation with mechanical ventilation [8]. In severe disease cases of EVALI where patients do not respond to invasive ventilation, veno-venous extracorporeal membrane oxygenation (VV-ECMO) is used to manage hypoxemia and hypercapnia. In rare circumstances where VV-ECMO has been utilized for an extended period with worsening clinical presentation, lung transplantation can be considered [2]. Another aspect of a treatment plan that is crucial in determining absolute resolution and good prognosis is the complete cessation of E-cigarettes use [8]. While there is a lack of long-term data on the prognosis of EVALI, clinicians should emphasize the importance of vaping cessation as E-cigarette use is an independent risk factor for cigarette smoking [9,10]. Perhaps, the increasing incidence of EVALI should provoke a public health discussion on regulating E-cigarettes and E-liquids [2].

## Conclusions

EVALI tends to present with a variety of symptoms that clinicians need to be aware of to make a conclusive differential diagnosis list. With less national attention to EVALI especially after the peak period of the disease, clinicians should continue to suspect EVALI especially in young patients who are considered relatively healthy with a history of E-cigarette use. Pneumomediastinum is a rare symptom seen with EVALI and should raise strong suspicion when noted in the radiographic imaging of a young patient who vapes. There is strong evidence that E-cigarette use is associated with cigarette initiation and current cigarette smoking among adolescents and youths. Clinicians should be aware of this correlation and begin early screening and counseling among adolescents and youths with the presence or absence of a vaping history.
